# Hydrogels with Ultrasound-Treated Hyaluronic Acid Regulate CD44-Mediated Angiogenic Potential of Human Vascular Endothelial Cells In Vitro

**DOI:** 10.3390/biom14050604

**Published:** 2024-05-20

**Authors:** Kelum Chamara Manoj Lakmal Elvitigala, Wildan Mubarok, Shinji Sakai

**Affiliations:** Department of Materials Engineering Science, Graduate School of Engineering Science, Osaka University, Toyonaka 560-8531, Osaka, Japan; kelum@cheng.es.osaka-u.ac.jp (K.C.M.L.E.); wildanmubarok@cheng.es.osaka-u.ac.jp (W.M.)

**Keywords:** endothelial cell network formation, hyaluronic acid, CD44 receptor, enzymatic crosslinking, composite hydrogel

## Abstract

The development of hydrogels that allow vascular endothelial cells to form capillary-like networks is critical for advancing tissue engineering and drug discovery. In this study, we developed hydrogels composed of phenolated hyaluronic acid (HA-Ph) with an average molecular weight of 490–159 kDa via sonication in an aqueous solution. These hydrogels were synthesized by the horseradish peroxidase-catalyzed crosslinking of phenol moieties in the presence of hydrogen peroxide and phenolated gelatin. The sonication-degraded HA-Ph (198 kDa) significantly enhanced the migration ability of human umbilical vein endothelial cells (HUVECs) on cell culture plates when added to the medium compared to the original HA-Ph (490 kDa) and less-degraded HA-Ph (312–399 kDa). In addition, HUVECs cultured on these hydrogels formed networks that did not occur on hydrogels made from the original HA-Ph. CD44 expression and PI3K gene expression, both markers related to angiogenesis, were 3.5- and 1.8-fold higher, respectively, in cells cultured on sonication-degraded HA-Ph hydrogels than in those cultured on hydrogels comprising the original HA-Ph. These results highlight the potential of hydrogels containing sonication-degraded HA-Ph for tissue engineering and drug-screening applications involving human vascular endothelial cells.

## 1. Introduction

Hyaluronic acid (HA), an endogenous glycosaminoglycan, is a crucial component of various tissues and is present in the extracellular matrix (ECM). It plays an important role in various physiological processes, such as angiogenesis, tissue regeneration, and wound healing [1,2,3]. In these physiological processes, the interactions of HA with the cell surface receptors CD44 and receptor for hyaluronan-mediated motility (RHAMM) are critical in modulating cellular functions such as adhesion, proliferation, and migration [4,5]. The ability of HA to interact with cells depends on its molecular weight [6]. HA can be classified into two main groups: high-molecular-weight HA (>500 kDa, HMWHA) and low-molecular-weight HA (10–500 kDa, LMWHA) [7]. LMWHA interacts distinctively with cell surface receptors such as CD44 and RHAMM in various cell types and induces unique intracellular signaling pathways [6]. The engagement of CD44 with LMWHA triggers a cascade of intracellular signaling and upregulates the synthesis of hyaluronidase, an enzyme responsible for degrading HA, resulting in the remodeling of the ECM [8].

Angiogenesis is an important process in both physiological and pathological contexts, including wound healing, tissue regeneration, and tumor growth [9,10]. Therefore, the molecular-weight-dependent regulation of HA on vascular endothelial cell behavior has been intensively studied [2,4,11,12]. However, gaps remain for on-demand control of the molecular weight of HA and its impact on the behavior of vascular endothelial cells. The effects of HA molecular weight and CD44 interactions on vascular endothelial cell network formation, which will provide valuable insights into the pro-angiogenesis process and the development of diseases such as cancer, cardiovascular diseases, and diabetic retinopathy, are particularly under-researched [13,14,15].

This study aimed to develop hydrogels incorporating LMWHA-Ph obtained by controlled sonication that allow vascular endothelial cells to form capillary-like networks through a process similar to angiogenesis in vivo, which is related to the expression of cell surface CD44, using human umbilical vein endothelial cells (HUVECs) in vitro. HUVECs have been widely used as model cells in angiogenesis studies to understand the behavior of vascular endothelial cells [16]. Hydrogels, which provide a three-dimensional matrix, are critical for mimicking the natural cellular environment, allowing a more accurate assessment of vascular endothelial cell behavior involving HA-regulated network formation.

**Figure 1:**
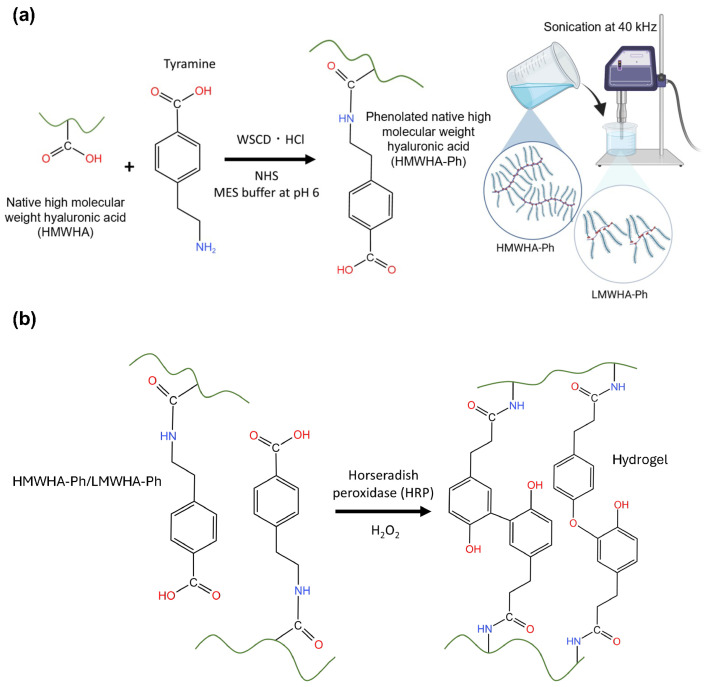
Schematic illustrations of (**a**) HA-Ph synthesis and degradation by sonication of HA-Ph solution to obtain LMWHA-Ph. (**b**) Hydrogel preparation method using HRP-mediated crosslinking of phenolated HMWHA-Ph or LMWHA-Ph in the presence of H_2_O_2_.

To obtain hydrogels composed of LMWHA, HMWHA possessing phenol moieties (HMWHA-Ph) was synthesized and then degraded by sonication to obtain LMWHA-Ph (Figure 1a). The sonication method was utilized to degrade HMWHA-Ph instead of enzymatic degradation using hyaluronidase or thermal degradation, which have been applied for the same purpose, owing to the difficulty of precise control of molecular weight [17]. Degradation by sonication allows finer control of the molecular weight of HA by adjusting the ultrasound frequency, intensity, and duration of exposure [18].

Hydrogels were obtained through horseradish peroxidase (HRP)-mediated crosslinking [19,20] of the phenol moieties introduced into HA (Figure 1b). Phenolated gelatin (gelatin-Ph) was also present in the hydrogels to support the adhesion and elongation of HUVECs, which was not accomplished via HA-Ph alone. The effectiveness of combining gelatin-Ph with a less cell-adhesive polymer-Ph to obtain hydrogels with superior cell adhesion and proliferation has been reported [21,22]. Several studies have demonstrated the potential of composite or hybrid hydrogels containing both HA and gelatin to mimic the ECM in the body [23,24,25], including their interaction with CD44 receptors [24]. The hydrogels obtained in this study will provide valuable insights into the mechanisms of angiogenesis and the role of ECM components in regulating this complex process, and they will advance tissue engineering and drug discovery involving vascular endothelial cells. It is hypothesized that hydrogels composed of sonication-degraded LMWHA-Ph will enhance the formation of capillary-like networks by HUVECs in vitro, potentially through upregulation of CD44 receptor expression and subsequent PI3K-mediated signaling pathways.

## 2. Materials and Methods

### 2.1. Materials

HA-Ph (3.5-Ph groups per 100 repeating units) and gelatin-Ph (4.1 × 10^−4^ mol-Ph/g) were prepared according to the previous protocols, and the phenol contents were determined based on the tyramine standard curve (Figure S1) [26,27]. Sodium hyaluronate (average molecular weight: 550 kDa, HA-LQ) was purchased from Kewpie (Tokyo, Japan) (molecular weight was measured as per the protocol described in Section 2.4. Molecular Weight Measurement). Tyramine hydrochloride, 3-(4-hydroxyphenyl)propionic acid, gelatin type B from bovine skin, and phalloidin-iFluor 488 (ab176759) were purchased from Chem-Impex (Wood Dale, IL, USA), Tokyo Chemical Industry (Tokyo, Japan), Sigma-Aldrich (St. Louis, MO, USA), and Abcam (Cambridge, UK), respectively. *N*-Hydroxysuccinimide, water-soluble carbodiimide hydrochloride, hyaluronidase, catalase from ovine, HRP, 31 *w*/*w*% hydrogen peroxide (H_2_O_2_) aqueous solution, and phosphate-buffered saline (PBS) containing 4 *w*/*w*% paraformaldehyde were purchased from FUJIFILM Wako Pure Chemical (Osaka, Japan).

### 2.2. Cell Culture

The HUEhT-1 (HUVECs modified with pIRES-hTERT-hygr) cell line was purchased from the RIKEN Cell Bank (Ibaraki, Japan). Cells were cultured in a humidified incubator with 5% CO_2_ at 37 °C using MCDB107 (Peptide Institute, Osaka, Japan) base medium supplemented with 10 ng/mL endothelial growth factor, 10 ng/mL basic fibroblast growth factor (Sigma-Aldrich), and 10 *v*/*v*% fetal bovine serum (FBS).

### 2.3. HA-Ph Degradation

PBS containing 2 *w*/*v*% HA-Ph (HA-Ph-0) was sonicated using an ultrasonic cleaner (LiebeWH, Shenzhen, China) operating at 40 kHz and 240 W at 50 °C for 5 min (HA-Ph-5), 10 min (HA-Ph-10), 30 min (HA-Ph-30), and 60 min (HA-Ph-60).

### 2.4. Molecular Weight Measurement

The polymer molecular weights were determined by high-performance liquid chromatography (HPLC) with respect to Pullulan standards using an intensity–time curve. The eluent flow rate in the column (LC-20AD; Shimadzu, Kyoto, Japan) was set to 0.7 mL/min at 25 °C.

### 2.5. Rheological Measurement

The viscoelastic properties of solutions containing 2 *w*/*v*% HA-Phs obtained after different sonication times were determined using a rheometer (HAAKE MARS III, Thermo Fisher Scientific, Waltham, MA, USA) with a cone plate (diameter: 35 mm) at 1% constant shear strain and a 1 mm gap between plates at 25 °C.

### 2.6. Hydrogel Preparation and Gelation Time

Hydrogels were prepared from PBS containing 2 *w*/*v*% and 1.5 *w*/*v*% HA-Ph-0 or 2 *w*/*v*% HA-Ph-30 and 10 U/mL HRP by exposure to 16 ppm air containing H_2_O_2_ for 30 min. A composite hydrogel (HA-Ph/gelatin-Ph) was prepared by mixing 0.1 *w*/*v*% gelatin-Ph with this solution. The gelation time was determined by adding 1 mL of the above polymer solutions into a 12-well plate and exposing it to 16 ppm air containing H_2_O_2_ while stirring with a magnetic bar. The gelation time was determined based on the swelling of the polymer solution.

### 2.7. Mechanical Property Measurement

Hydrogels were prepared by exposing air containing 16 ppm H_2_O_2_ to PBS containing 0.1 *w*/*v*% gelatin-Ph, 2 *w*/*v*% HA-Ph-0 to HA-Ph-60, and 10 U/mL HRP in a 6-well plate (1 mL/well) for 30 min (diameter 35 mm and height 3 mm). The stiffness of these hydrogels was measured using a material tester (EZ-SX, Shimadzu, Kyoto, Japan) equipped with a load cell having a sensitivity of 5 N. An 8 mm diameter probe was used to apply localized compression to the hydrogel sheet during the compression test at a compression speed of 6 mm/s. Young’s modulus was calculated from data obtained in the strain range of 1% to 10%.

### 2.8. Diphenol Formation

PBS containing 2 *w*/*v*% HA-Ph-0 to HA-Ph-60 and 10 U/mL HRP was poured into a 96-well plate (200 μL/well) and exposed to air containing 16 ppm H_2_O_2_. Diphenol bond formation was analyzed using a fluorescence plate reader (Molecular Devices, San Jose, CA, USA). Fluorescence emission intensity at 420 nm was measured at an excitation wavelength of 310 nm.

### 2.9. Impact of Sonicated HA-Ph Solutions on Cellular Dynamics

#### 2.9.1. Cell Migration

HUEhT-1 cells were cultured in 6-well plates until they reached confluence. A scratch was created using a pipette tip. After scratching, the growth medium was replenished with a growth medium containing 0.1 *w*/*v*% HA-Ph-0 to HA-Ph-60. Cell migration speed, defined as the scratch area covered by the cells over time, was monitored using a cell culture monitoring system (CM20, Olympus, Tokyo, Japan).

#### 2.9.2. Cell Proliferation

HUEhT-1 cell proliferation was determined via cell doubling time. Cells were cultured in a 24-well plate at 5 × 10^2^ cells/cm^2^ using MCDB107 growth medium containing 0.1 *w*/*v*% HA-Ph-0 to HA-Ph-60 and monitored using the cell culture monitoring system to calculate the doubling time.

### 2.10. Cell Adhesion and Morphology on Hydrogel

Hydrogels were prepared from solutions containing 10 U/mL HRP, 0.1 *w*/*v*% gelatin-Ph, and 1.5 or 2 *w*/*v*% HA-Ph-0 or 2 *w*/*v*% HA-Ph-30 in 6-well plates following the protocol outlined in Section 2.6. Hydrogel Preparation and Gelation Time, and cells at 5 × 10^3^ cells/cm^2^ were seeded on the hydrogels. Before cell seeding, the residual H_2_O_2_ on the hydrogels was degraded by overnight incubation in MCDB107 medium containing 1 mg/mL catalase. After 48 h of culture, F-actin and nuclei were stained with the phalloidin iFluor488 reagent and CellStain DAPI (Dojindo, Kumamoto, Japan), respectively. These stains facilitated the analysis of cell morphological parameters, specifically cell area and aspect ratio (the ratio between cell length and width). The morphological parameters were analyzed based on fluorescence images captured using a fluorescence microscope (Model BZ-9000, Keyence, Osaka, Japan) and ImageJ software (Version 1.53f, NIH, Bethesda, MD, USA).

### 2.11. Network Formation

HUEhT-1 cell network formation on the prepared hydrogels was analyzed as described in Section 2.6. Hydrogel Preparation and Gelation Time. Then, CD44 receptor-blocked and non-blocked HUEhT-1 cells at 4 × 10^4^ cells/cm^2^ were cultured on the hydrogels using MCDB107 medium supplemented with 2 *v*/*v*% FBS, 10 ng/mL endothelial growth factor, and 10 ng/mL basic fibroblast growth factor for 16 h. Before cell seeding, the remaining H_2_O_2_ was degraded according to the method described in 2.10. Cell Adhesion and Morphology on Hydrogel. A fluorescence microscope and a cell culture monitoring system were used to analyze network formation.

### 2.12. Flow Cytometry

HuEhT-1 cells were collected from the hydrogel via 3 h incubation in MCDB107 medium containing 0.1 *w*/*v*% hyaluronidase (FUJIFILM Wako Pure Chemicals). Next, the cells were incubated with FCblock (BD Bioscience, San Jose, CA, USA) reagents to block non-specific binding sites. Subsequently, cells were washed with PBS and incubated PBS containing APC-conjugated mouse CD44 antibody (1:300) for 30 min at 4 °C. After 30 min of incubation, the cells were washed twice with PBS and analyzed using a BD Accuri C6 flow cytometer (BD Biosciences).

### 2.13. Real-Time Quantitative Polymerase Chain Reaction (PCR) Analysis for PI3K and Hypoxia-Inducible Factor (HIF)-1 Expression

HUEhT-1 cells were collected from the hydrogel as described in Section 2.12. Flow Cytometry. For comparison, the cells cultured in the dish were trypsinized. Total RNA was isolated from cells using an RNA isolation kit (Takara, Shiga, Japan) according to the manufacturer’s protocol. Reverse transcription was performed using the PrimerScript RT Master Mix reagent kit (Takara) according to the manufacturer’s protocol. PI3K and HIF-1 gene expression were quantified via real-time polymerase chain reaction (RT-PCR) using the TB Green Master Kit (Takara) normalized to the expression of the GAPDH gene with the delta Ct method.

### 2.14. Statistical Analysis

Statistical analyses were performed using Microsoft Excel 2019 version 1808 (Microsoft Corp., Redmond, WA, USA). A one-way analysis of variance was used to determine statistical differences between experimental conditions. Significant differences were identified using Tukey’s honestly significant difference (HSD) and the post hoc *t*-test; *p* < 0.05 was considered statistically different.

## 3. Results and Discussion

### 3.1. Viscoelastic Properties, Molecular Weight, and Hydrogel Characterization

**Figure 2:**
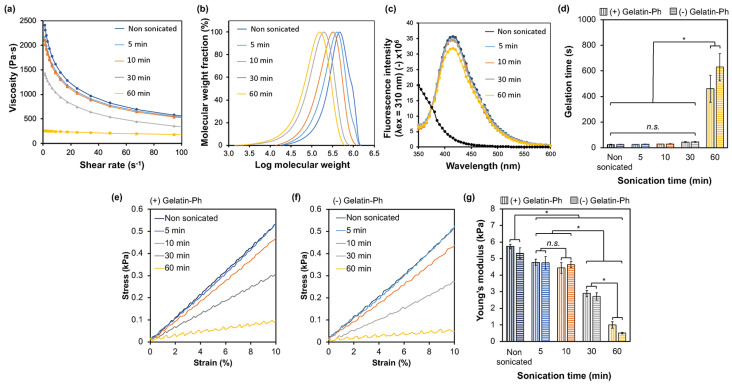
Effect of sonication time on the (**a**) viscosity of HA-Ph solution and (**b**) molecular weight of HA-Ph. The stability of phenol groups with sonication time represents the fluorescence emission of the (**c**) diphenol bond at excitation at 310 nm. The effect of sonication time on the mechanical properties of HA-Ph hydrogels with and without gelatin-Ph is represented as (**d**) gelatin time and (**g**) Young’s modulus calculated based on the (**e**,**f**) stress−strain curve at 1% to 10% strain. Error bars represent the standard deviation (*n* = 5) for Young’s modulus and (*n* = 3) for gelation time. * *p* < 0.05, n.s.: no significant difference (*p* > 0.05) in Tukey’s HSD.

The possibility of HA-Ph degradation by sonication was evaluated by measuring the change in viscosity of the sonicated HA-Ph solutions and the molecular weight of HA-Ph in the solutions. As shown in Figure 2a, the time-dependent degradation of HA-Ph was indicated by the decrease in the viscosity of the solutions with increasing sonication time; 30 and 60 min of sonication resulted in a 39% and 68% reduction in viscosity, respectively, compared with that of the non-sonicated solution. The average molecular weight of HA-Ph in the sonicated solutions, determined via HPLC analysis (Figure 2b and Figure S3), decreased from 490 kDa (HA-Ph in the non-sonicated solution) to 198 and 159 kDa after 30 and 60 min of sonication, respectively. These results demonstrate that HA-Ph is degradable by sonication, similar to unmodified HA. Ultrasound treatment causes HA chains to break into shorter fragments, mainly through the breakage of glycosidic bonds [18].

The phenol moieties were introduced to obtain hydrogels through HRP-mediated oxidative crosslinking. Phenols can be oxidized by sonication [28,29]. Therefore, the remaining unoxidized phenol moieties were analyzed, HA-Phs, in the sonicated solutions by measuring diphenol formation through HRP-mediated crosslinking. The fluorescence emission of diphenol at 420 nm for the HA-Ph solutions sonicated for 5–30 min showed no changes, whereas 60 min of sonication showed a lower fluorescence emission compared to that detected for the non-sonicated HA-Ph solution. This result indicates that sonication causes the oxidation of the phenolic moieties of HA-Ph, but the degree of oxidation is not as high with treatments lasting less than 60 min under the conditions applied in this study (Figure 2c).

Next, the gelation time and mechanical properties of the hydrogels formed from the solutions obtained through HRP-mediated crosslinking were evaluated, which are affected by the molecular weight of the polymers [30] and the density of crosslinkable phenol moieties [26,31]. These are crucial parameters for hydrogel sheet fabrication in in vitro cell culture studies. The gelation time increased significantly with the sonication time. A longer average gelation time of 600 s was obtained with a 60 min sonicated HA-Ph solution, which was nearly 10 times higher than that for all the other sonication times (Figure 2d) (*p* < 0.05). The hydrogel stiffness (as measured by Young’s modulus calculated from data obtained in the strain range of 1% to 10% (Figure 2e,f)) decreased with increasing sonication time (Figure 2g). The hydrogels obtained from the HA-Ph solutions sonicated for 5 and 10 min exhibited a negligible change in stiffness (*p* = 0.4), whereas those from the solutions sonicated for 30 and 60 min decreased Young’s modulus to 49% and 90%, respectively, compared to that of the hydrogel obtained from the non-sonicated HA-Ph solution (*p* < 0.05). These results demonstrate that the hydrogelation time and mechanical properties of the hydrogels obtained from sonicated HA-Ph solutions can be controlled by varying the sonication time.

The effect of adding 0.1 *w*/*v*% gelatin-Ph to the HA-Ph solutions on the gelation time and mechanical properties of the hydrogels was also evaluated because the incorporation of gelatin-Ph is necessary for cell adhesion and growth on HA-Ph hydrogels [27]. As shown in Figure 2d,g, the addition had a negligible effect on the gelation time and hydrogel stiffness within 30 min of sonication. This result indicates that the hydrogelation rate and mechanical properties were mainly due to HA-Ph at 0.1 *w*/*v*% gelatin-Ph under these conditions.

### 3.2. Influence of Sonicated HA-Ph Solutions on Cell Migration and Proliferation

**Figure 3:**
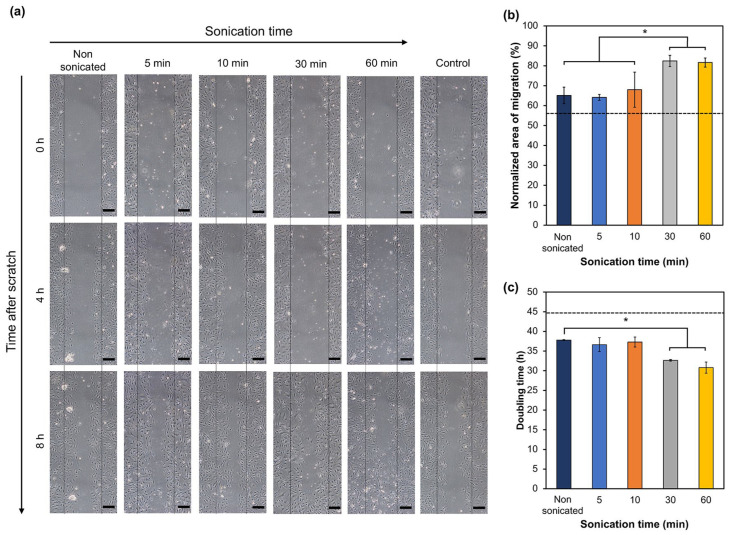
Impact of medium containing degraded HA-Ph on HUEhT-1 cell migration and proliferation. (**a**) Time-lapse images captured at 0, 4, and 8 h after creating a scratch wound (scale bar 50 μm). (**b**) Quantitative analysis of the normalized area of cell migration as a percentage. Error bars represent the standard deviation (*n* = 3). (**c**) Doubling time of HUEhT-1 cells as a function of HA-Ph solution sonication time. Error bars represent the standard deviation (*n* = 2). Cells cultured without adding HA-Ph into the medium were considered the control, and the values are represented as the dashed line in the graphs. * *p* < 0.05, n.s.: no significant difference (*p* > 0.05) Tukey’s HSD.

Low-molecular-weight HA fragments enhance endothelial cell migration [2,32] and proliferation [33,34]. Therefore, the evaluation focused on whether HA-Ph degraded by sonication can promote vascular endothelial cell migration and proliferation, similar to unmodified HA. As shown in Figure 3, HUEhT-1 cells cultured in the presence of HA-Ph-30 and HA-Ph-60 showed significantly enhanced migration speed by 21% and 20%, respectively, compared to those cultured in the presence of non-sonicated HA-Ph (HA-Ph-0) (*p* < 0.05) (Figure 3a,b). Proliferation was enhanced by HA-Ph-30 and HA-Ph-60, as shown in Figure 3c. The cells treated with HA-Ph-30 and HA-Ph-60 showed decreased doubling time by approximately 27% and 31%, respectively, compared to that of untreated cells (*p* < 0.05) (Figure 3c).

These results demonstrate that low-molecular-weight HA-Ph obtained by sonication-mediated degradation can enhance the migration and proliferation of vascular endothelial cells, similar to non-modified HA. Slevin et al. have reported that the interaction between LMWHA and vascular endothelial cells enhances cell migration speed via the activation of extracellular-regulated kinase 1/2 (ERK1/2) [2]. Low-molecular-weight HA regulates vascular endothelial cell proliferation through interactions with cell receptors such as CD44 [35]. Clustering of CD44 on the cell surface was proposed to enhance the production of vascular endothelial growth factor (VEGF), promoting EC proliferation [34].

### 3.3. Cell Adhesion and Morphology on Hydrogels

Understanding the adhesion of HUEhT-1 cells is essential to elucidating their ability to form networks, particularly when interacting with HA of different molecular weights. Based on the above results for the formation of hydrogels in a short time (Figure 2d,g) and the enhancement of migration with a smaller decrease in cell growth (Figure 3), hydrogels composed of 2 *w*/*v*% HA-Ph-30 and 0.1 *w*/*v*% gelatin-Ph (HA-Ph-30 hydrogel) were used to evaluate the effect of the incorporation of degraded HA-Ph through sonication on the behavior of vascular endothelial HUEhT-1 cells. Cellular adhesion is governed by substrate stiffness [36]. As shown in Figure 2g, the 2 *w*/*v*% HA-Ph-0 and 2 *w*/*v*% HA-Ph-30 hydrogels exhibited different stiffness values. Therefore, to mitigate the impact on hydrogel stiffness and investigate the effect of the HA-Ph molecular weight on cellular adhesion, 1.5 *w*/*v*% HA-Ph-0 hydrogel (Young’s modulus: 2.9 kPa) was utilized, which also has nearly the same stiffness as 2 *w*/*v*% HA-Ph-30 hydrogel (Young’s modulus: 2.7 kPa, Figure 2g).

**Figure 4:**
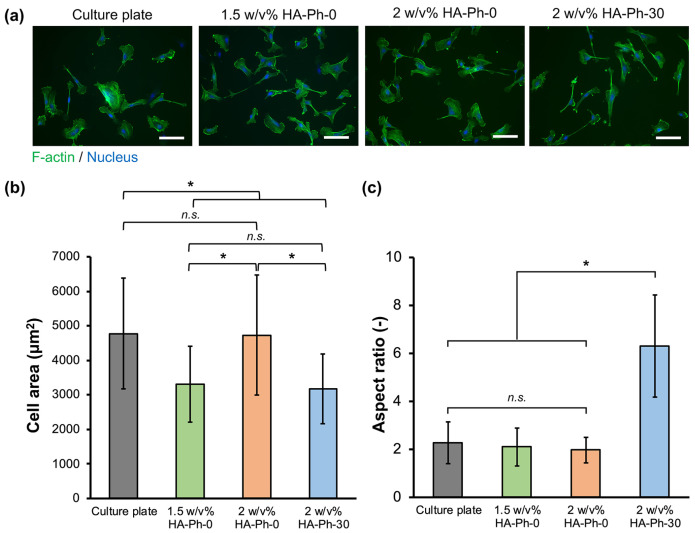
Evaluation of HUEhT-1 cell adhesion and morphology on the hydrogels composed of 2 *w*/*v*% or 1.5 *w*/*v*% HA-Ph-0 and 2 *w*/*v*% HA-Ph-30. (**a**) Fluorescence micrographs of the HUEhT-1 cells on hydrogels stained with phalloidin iFluor-488 (F-actin) and DAPI (nucleus). Scale bars: 100 μm. (**b**) Area and (**c**) aspect ratio of cells on hydrogels (*n* ≥ 40). * p < 0.05, n.s.: no significant difference (*p* > 0.05), Tukey’s HSD. Error bars represent the standard deviation.

As shown in Figure 4, HUEhT-1 cells cultured on the 2 *w*/*v*% HA-Ph-0 hydrogel showed a similar shape (a), cell area (b), and aspect ratio (c) to those on a cell culture plate. The cells on the 1.5 *w*/*v*% HA-Ph-0 hydrogel showed a similar shape and aspect ratio as those on the 2 *w*/*v*% HA-Ph-0 hydrogel and culture plate, but with a smaller cell area (*p* < 0.05). The cells on the 2 *w*/*v*% HA-Ph-30 hydrogel showed a similar cell area to those on the 1.5 *w*/*v*% HA-Ph-0 hydrogel (*p* = 0.9) but showed an approximately 3-fold larger aspect ratio (*p* < 0.05). As consistent with our previous studies, LMWHA-Ph induced significant cellular elongation [37,38]. A possible mechanism for cell elongation on the HA-Ph-30 hydrogel is the epithelial-to-mesenchymal transition [39], which involves morphological changes of cells to an elongated spindle-like morphology. A previous study by Pang et al. also reported that LMWHA could induce cell elongation [40].

### 3.4. CD44-Mediated HUEhT-1 Cell Network Formation

**Figure 5:**
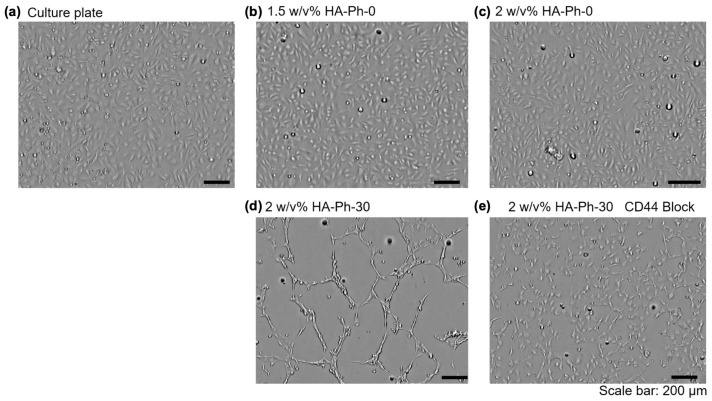
HUEhT-1 cell network formation assay on the (**a**) culture plate, (**b**) hydrogels composed of 1.5 *w*/*v*% HA-Ph-0, (**c**) 2 *w*/*v*% HA-Ph-0, (**d**) 2 *w*/*v*% HA-Ph-30, and (**e**) 2 *w*/*v*% HA-Ph-30 blocked with CD44. Cells were observed after 13 h of culture.

Next, the network formation of HUEhT-1 cells on hydrogels with the same composition of HA-Phs as described in Section 3.3. Cell Adhesion and Morphology on Hydrogels was investigated. HUEhT-1 cells only formed a visible network-like structure in the 2 *w*/*v*% HA-Ph-30 hydrogel (Figure 5d and Movie S1). In contrast, the cells cultured on the culture plate (Figure 5a) and hydrogels with 1.5 *w*/*v*% (Figure 5b) and 2 *w*/*v*% HA-Ph-0 (Figure 5c) exhibited no discernible network formation. This difference underscores the pivotal role of the molecular weight of HA-Ph, particularly that of HA-Ph-30, in vascular endothelial cell network formation. A targeted approach was used to elucidate the specific interactions between the CD44 receptors and HA-Ph-30. The CD44 receptors on HUEhT-1 cells were selectively blocked using an anti-CD44 antibody before cell seeding onto the 2 *w*/*v*% HA-Ph-30 hydrogel (Figure S5). Cells in which the CD44 receptors were blocked showed no network formation on the 2 *w*/*v*% HA-Ph-30 hydrogel (Figure 5e), whereas non-blocked cells exhibited robust network-like structures (Figure 5d). HA oligomers obtained via enzymatic degradation promote network formation by activating ICAM-1 and VCAM-1 expression [34]. The interaction of HA oligomers with CD44 receptors enhanced the production of VEGF, an essential growth factor in angiogenesis [5]. Therefore, the suppression of network formation under CD44-blocked conditions may be due to the regulation of the necessary signaling pathways for the secretion of necessary growth factors. Also, LMWHA-Ph interaction with CD44 receptors could activate γ-adducin, which plays a role in network formation [41].

### 3.5. HA-Ph Molecular Weight Modulates the Expression of CD44 Receptors

**Figure 6:**
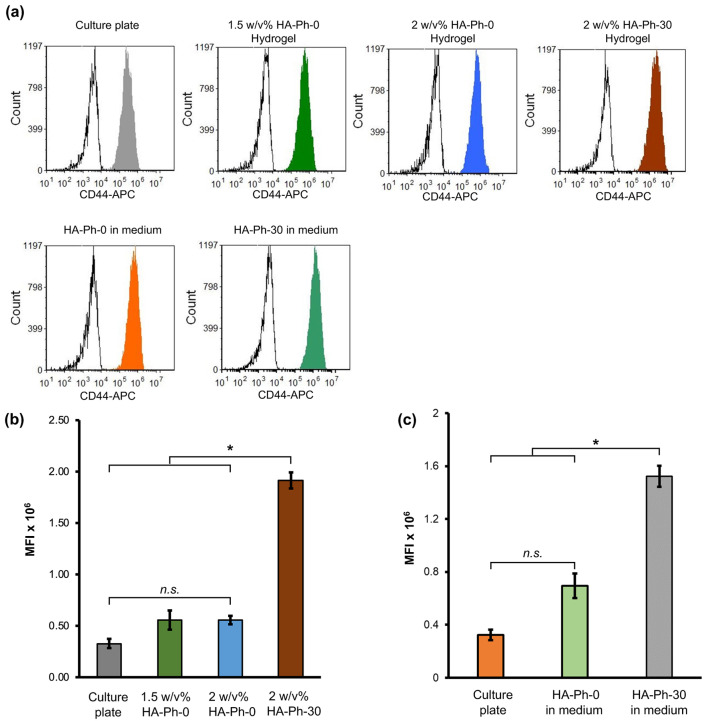
Flow cytometry analysis of HUEhT-1 cells. (**a**) Representative flow cytometry histogram of HUEhT-1 cells cultured on the hydrogels and well plate (medium containing different molecular weights of HA-Ph). The quantitative mean fluorescence intensity (MFI) values of HUEhT-1 cells cultured on the (**b**) hydrogel composed of 2 *w*/*v*% or 1.5 *w*/*v*% HA-Ph-0 and 2 *w*/*v*% HA-Ph-30 and (**c**) culture plate (culture medium consisting of 0.1 *w*/*v*% HA-Ph-0 and HA-Ph-30). Error bar: standard deviation (*n* > 1000 cells). * *p* < 0.05, n.s.: no significant difference (*p* > 0.05), Tukey’s HSD.

Based on flow cytometry analysis, CD44 expression was notably influenced by the molecular weight of the HA-Phs, as shown in Figure 6. CD44 expression in HUEhT-1 cells cultured on a cell culture plate and 1.5 *w*/*v*% and 2 *w*/*v*% HA-Ph-0 hydrogels decreased by 83, 71, and 71%, respectively, compared to those cultured on the 2 *w*/*v*% HA-Ph-30 hydrogel (*p* < 0.05) (Figure 6a,b). These results are consistent with our recent study and those of Khanmohammadi et al., who showed that LMWHA-immobilized gelatin-based hydrogels increased endothelial cell motility and CD44 expression [42,43].

The soluble forms of HA-Phs were also used to elucidate the effect of the molecular weight of the HA-Phs on CD44 expression (Figure 6a,c) by incorporating HA-Ph-0 and HA-Ph-30 into the cell culture medium at a concentration of 0.1 *w*/*v*%. The mean fluorescence intensity (MFI) for the untreated cells and 0.1 *w*/*v*% HA-Ph-0-treated cells showed a decrease of approximately 78% and 54%, respectively, compared to the 0.1 *w*/*v*% HA-Ph-30-treated cells (*p* < 0.05). These results demonstrate that both the crosslinked and soluble forms of HA-Ph-30 enhance CD44 expression in HUEhT-1 cells. This result is in accordance with a previous report on human cerebral microvascular ECs (HCMVECs) in the presence of both soluble and crosslinked HA [11].

### 3.6. Effect of HA-Ph-CD44 Interaction on PI3K and HIF-1 Gene Expression

**Figure 7:**
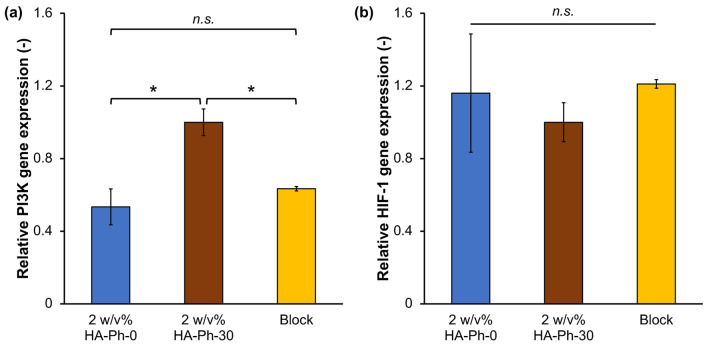
Relative gene expression levels of (**a**) PI3K and (**b**) HIF-1 in HUEhT-1 cells cultured in 2 *w*/*v*% HA-Ph-0 and 2 *w*/*v*% HA-Ph-30 hydrogels. Relative Ct values were calculated with respect to the cells cultured on the 2 *w*/*v*% HA-Ph-30 hydrogel. Error bar: standard deviation (*n* = 2). * *p* < 0.05, n.s.: no significant difference (*p* > 0.05), Tukey’s HSD.

PI3K and HIF genes play a significant role in angiogenesis [44,45,46]. Therefore, the effect of the HA-Phs obtained by sonication-mediated degradation on PI3K gene expression in HUEhT-1 cells cultured on hydrogels was analyzed. The cells cultured on the 2 *w*/*v*% HA-Ph-30 hydrogel showed 1.5-fold and 1.8-fold higher PI3K gene expression than those cultured on the same hydrogel with CD44 blocked and the 2 *w*/*v*% HA-Ph-0 hydrogel (*p* < 0.05), respectively, indicating that the interaction of HA-Ph-30 with CD44 receptors may increase PI3K gene expression (Figure 7a). Hypoxia-inducible factor 1 (HIF-1) is a key gene involved in angiogenesis under hypoxic conditions [47]. Notably, no significant differences were observed in HIF-1 gene expression across all tested conditions (*p* > 0.05) (Figure 7b). These findings suggest that hypoxic conditions did not primarily govern network formation in HUEhT-1 cells but were likely due to the interaction between LMWHA and CD44, as evidenced by the significant variations observed in PI3K gene expression. This result supports the previous findings, which reported the influence of the PI3K gene in angiogenesis and its activation through various signaling pathways initiated by the interaction of CD44 and HA [44,45,46].

## 4. Conclusions

In this study, we investigated the role of HA-Ph molecular weight, obtained by sonication of HA-Ph solutions, in vascular endothelial cell network formation within a hydrogel prepared by HRP-mediated crosslinking. The degree of HA-Ph degradation was tuned by controlling the sonication time at a constant frequency. Human vascular endothelial cells exhibited distinct migration speeds and proliferation depending on the degree of degradation. The degraded HA-Ph present in the hydrogel promoted the formation of the HUEhT-1 cell network via CD44 interactions and elevated the expression of PI3K. These results indicate that the sonication-mediated degradation of HA-Ph plays a crucial role in vascular endothelial cell behavior. Therefore, these results provide a promising avenue for fabricating hydrogels for in vitro vascular endothelial cell studies. Future research should explore the CD44-LMWHA-Ph interaction in specific signaling pathways related to network-like structure formation in endothelial cells. This study provides valuable insights into the angiogenic potential of human vascular endothelial cells mediated by the CD44-LMWHA-Ph interaction. Notably, we have focused exclusively on the LMWHA-Ph-CD44 receptor interaction. Other hyaluronan receptors, such as RHAMM, also interact with LMWHA and play significant roles in mediating the cellular responses to HA. Our study did not address the potential effects of the interaction of LMWHA-Ph with other receptors, such as RHAMM, which is a limitation of our current research. Future studies should explore the role of other receptor interactions with the LMWHA obtained via sonication to fully understand the mechanisms of angiogenesis.

## Data Availability

Data are available upon reasonable request to the corresponding author.

## Supplementary Materials

The following supporting information can be downloaded at: https://www.mdpi.com/article/10.3390/biom14050604/s1, Figure S1: UV–Vis absorbance spectrum of (a) Na-HA, HA-Ph, and (b) gelatin, gelatin-Ph; (c) tyramine hydrochloride standard curve was used to determine the phenol content in each HA-Ph and gelatin-Ph; Figure S2: ^1^H NMR spectroscopy of (a) Na-HA, HA-Ph, and (b) gelatin, gelatin-Ph; Figure S3: Intensity–time curve used for molecular weight distribution calculations after sonication of HA-Ph solutions for 5–60 min. Average molecular weights and molecular weight distribution calculations were conducted with respect to Pullulan standards. PBS was used as the eluent, and the flow rate was set to 0.7 mL/min at 25 °C; Figure S4: Effect of the sonication time and the temperature on the oxidation of phenol groups present in the HA-Ph. UV–Vis absorbance spectrum of phenol groups at 275 nm after (a) different sonication times at a constant temperature and (b) incubation for 5–60 min at 50 °C without sonication. (c) Diphenol formation between phenol moieties after incubation at 50 °C for 5–60 min. Additionally, 0 min samples were maintained at 4 °C in UV–Vis and diphenol formation experiments; Figure S5: HUEhT-1 cell network formation assay on the hydrogels composed of 2 *w*/*v*% HA-Ph-30. Phase contrast and fluorescence images of HUEhT-1 network formation after 16 h of culture on the hydrogel under (a) CD44 receptor non-blocked and (b) CD44 receptor block (red: APC-CD44 antibody) conditions; Movie S1: HUEhT-1 network formation.
