# Supplementary material for: Hydrogels with Ultrasound-Treated Hyaluronic Acid Regulate CD44-Mediated Angiogenic Potential of Human Vascular Endothelial Cells In Vitro
Source: Biomolecules. 2024 May 20;14(5):604. doi: 10.3390/biom14050604 (PMC11118219; doi:10.3390/biom14050604)
Supplement: Supplementary file 1 [file biomolecules-14-00604-s001.zip › Supplementary Information_Biomolecules_Revised.pdf]

biomolecules

MDPI

Supplementary Information

Hydrogels with ultrasound-treated hyaluronic acid regulate CD44-mediated angiogenic potential of human vascular endothelial cells *in vitro*

Kelum Chamara Manoj Lakmal Elvitigala, Wildan Mubarok, and Shinji Sakai*

Department of Materials Engineering Science, Graduate School of Engineering Science, Osaka University, Toyonaka, Osaka, 560-8531, Japan.
*Correspondence: sakai@cheng.es.osaka-u.ac.jp

Supplementary Methods

1. Preparation of Phenolated HA (HA-Ph)

Na-HA (1.87 g) was dissolved in 500 mL of 0.1M MES buffer solution (pH 6). Then, tyramine hydrochloride (6.95 g), water-soluble carbodiimide (WSCD.HCl, 2.76 g), and N-hydroxysulfoccinimide (NHS, 1.57 g) were added to the above solution and stirred for 20 hours. The resulting HA-Ph solution was dialyzed against deionized water until no peak was detected at 275 nm in the deionized water, indicating that no tyramine residue was left. Phenolation was determined based on the peak at 275 nm and 6-8 ppm using UV spectroscopy and NMR, respectively **(Figure S1a and Figure S2a)**.

2. Preparation of Phenolated Gelatin (Gelatin-Ph)

Gelatin type B (20 g) was dissolved in 500 mL of dimethylformamide (DMF) buffer solution (pH 4.2). Then, 3-(4-hydroxyphenyl) propionic acid (6.64 g), WSCD.HCl (7.6 g), and NHS (6.4 g) were added to the above solution and stirred for 20 hours. Following the 20h reaction, unreacted 3-(4-hydroxyphenyl) propionic acid was eliminated from the mixture by dialyzing the solution in deionized water. Phenolation was determined based on the peak at 275 nm and 6-8 ppm using UV spectroscopy and NMR, respectively **(Figure S1b and Figure S2b)**.

3. Polymer characterization

3.1 UV-Vis

The phenolated and non-phenolated polymers were dissolved in PBS at a concentration of 0.1 w/v%, and the absorbance was measured at 275 nm to determine the phenol content in the polymer backbone **(Figure S1-a-c)**. Tyramine hydrochloride was used to prepare a calibration curve, as shown in **Figure S1e**.

3.2 ^{1}H NMR spectroscopy

The phenolation of all three polymers was determined using NMR spectroscopy, as shown in **Figure S2**. Phenolated and non-phenolated polymers were dissolved in D_2O at

a concentration of 0.1 w/v%. Then, samples were characterized using NMR spectroscopy (JNM ECS-400, JEOL, Tokyo, Japan) operating at 400 Hz and 25°C

4. Determination of sonication time on the oxidation of phenol moieties of HA-Ph

The effect of sonication energy on the oxidation of phenol moieties of HA-Ph was determined using UV-Vis and diphenol formation. First, PBS containing 0.1 w/v% HA-Ph solution was subjected to sonication for 5-60 min at 50°C and analyzed by UV-Vis to determine the absorbance of phenol groups at 275 nm **(Figure S4a)**. The same experiment was conducted to check the effect of temperature by determining the absorbance of phenol groups at 275 nm after incubation at 50°C for 5, 10, 30, and 60 min without sonication **(Figure S4b)**. Before incubation, samples were maintained at 4°C. The effect of temperature on the phenol group oxidation was further confirmed by fluorescence-based diphenol formation analysis. PBS containing 2 w/v% HA-Ph was incubated at 50°C for 5-60 min and mixed with 10 U/mL HRP. Then, exposed to air containing 16 ppm H_2O_2 for 30 min and checked the diphenol formation by the fluorescence emission at 420 nm following the excitation at 310 nm **(Figure S4c)**.

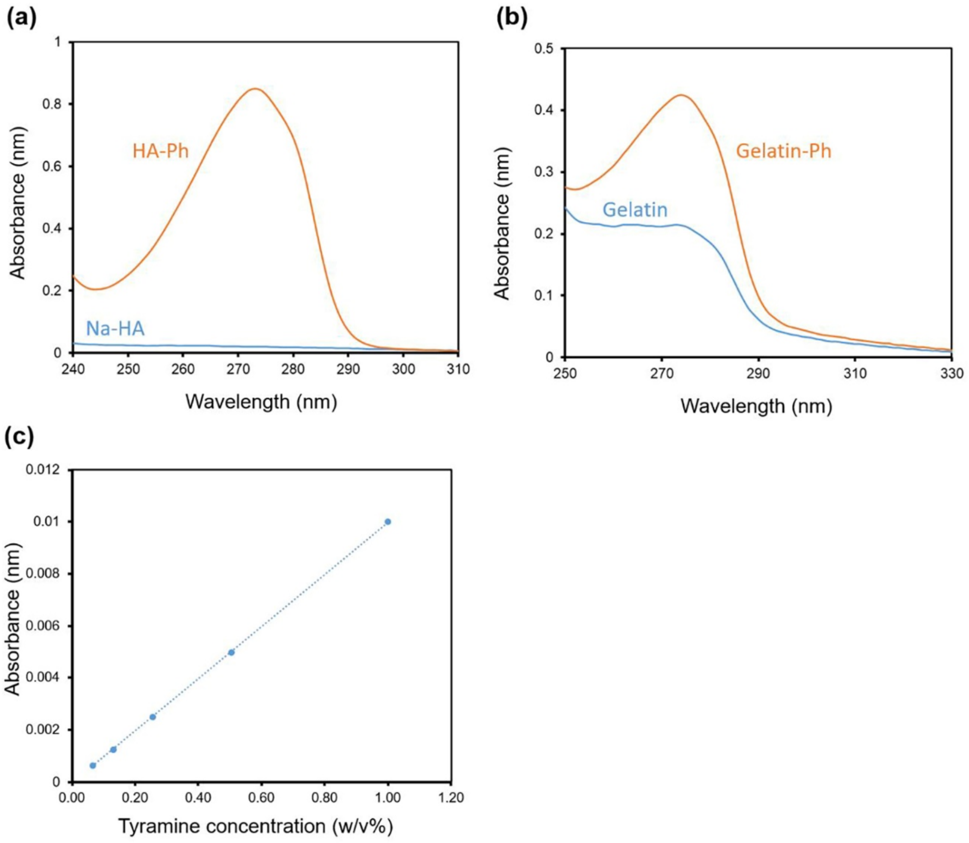

Figure S1. UV-Vis absorbance spectrum of (a) Na-HA, HA-Ph and (b) Gelatin, Gelatin-Ph (c) tyramine hydrochloride standard curve was used to determine the phenol content in each HA-Ph and Gelatin-Ph.

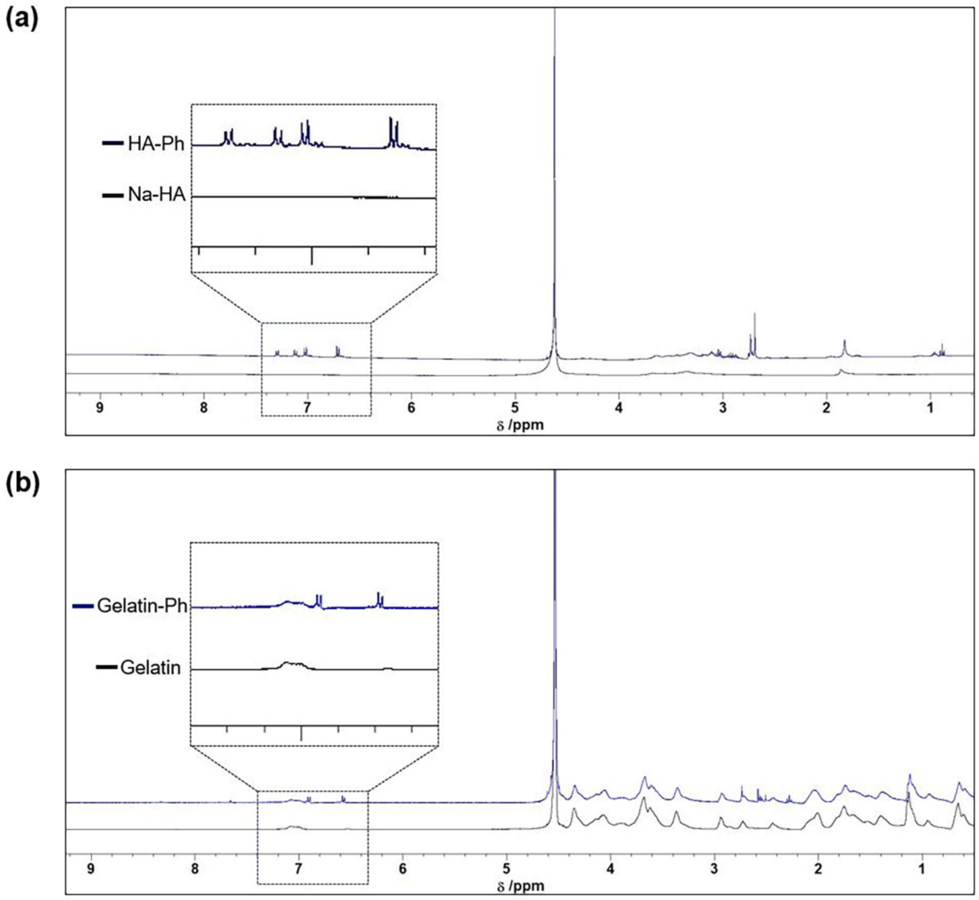

Figure S2. ^{1}H NMR spectroscopy of the (a) Na-HA, HA-Ph and (b) Gelatin, Gelatin-Ph.

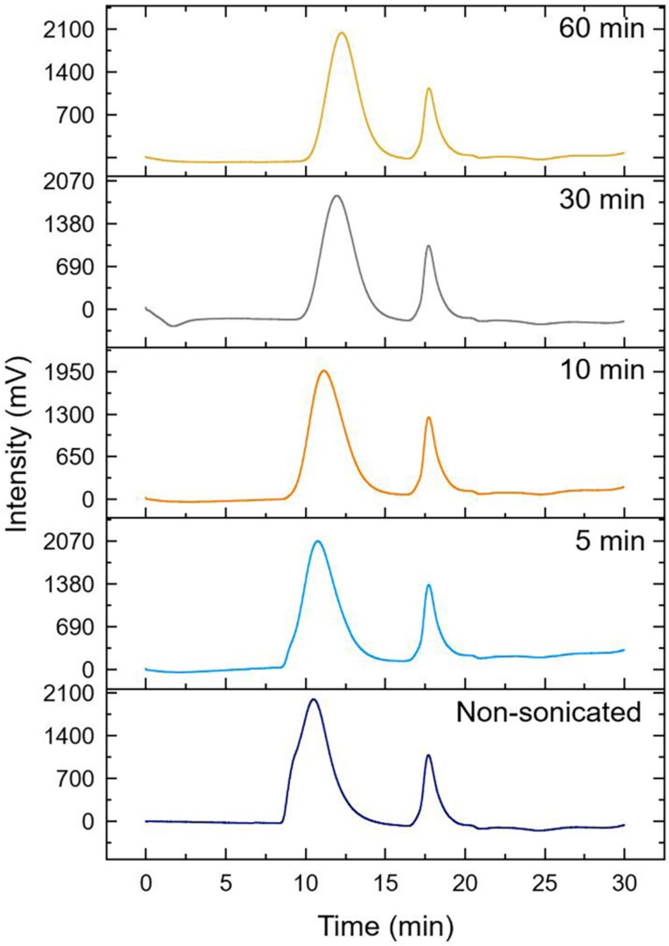

Figure S3. Intensity-time curve used for molecular weight distribution calculations after sonication of HA-Ph solutions for 5-60 min. Average molecular weights and molecular weight distribution calculations were done with respect to Pullulan standards. PBS was used as the eluent, and the flow rate was set to 0.7 ml/min at 25°C.

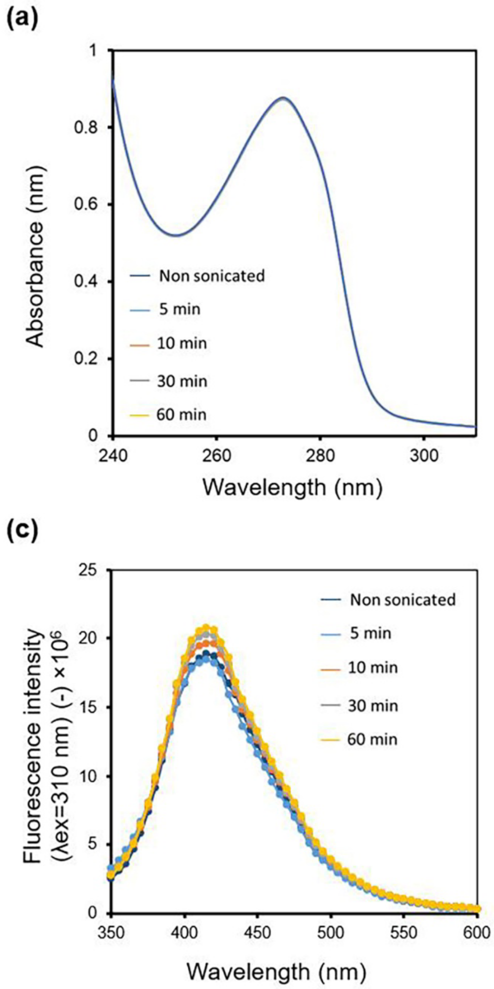

Figure S4. Effect of the sonication time and the temperature on the oxidation of phenol groups present in the HA-Ph. UV-Vis absorbance spectrum of phenol groups at 275 nm after (a) different sonication times in a constant temperature and (b) incubation for 5-60 min at 50 °C without sonication. (c) Diphenol formation between phenol moieties after incubation at 50 °C for 5-60 min. 0 min samples were maintained at 4 °C in UV-Vis and diphenol formation experiments.

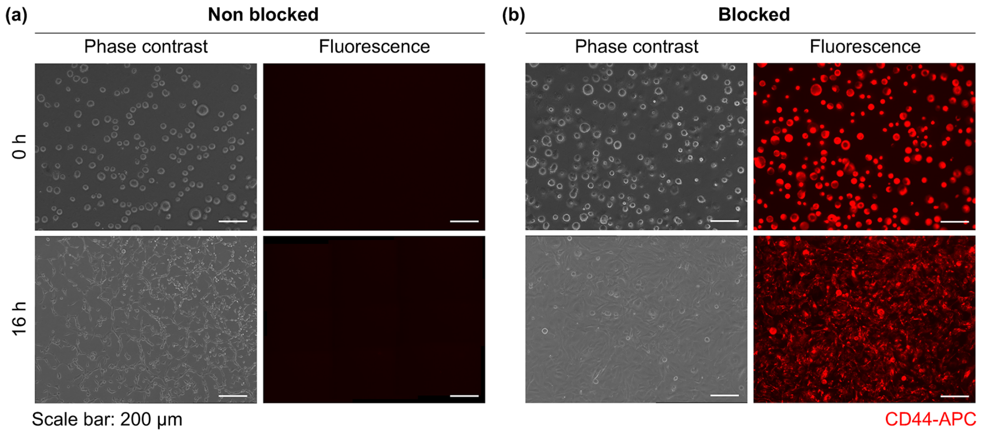

Figure S5. HUEhT-1 cell network formation assay on the hydrogels composed of 2 w/v% HA-Ph-30. Phase contrast and fluorescence images of HUEhT-1 network formation after 16h of culture on the hydrogel under (a) CD44 receptor non blocked and (b) CD44 receptor block (red: APC-CD44 antibody) conditions.
